# Association between the apolipoprotein B/albumin ratio and the risk of kidney stones in adults

**DOI:** 10.3389/fphys.2026.1892141

**Published:** 2026-08-05

**Authors:** Leilei Ke, Haibo Qin, Fengcheng Wang, Tong Yin, Xudong Shen, Zongyao Hao

**Affiliations:** 1 Department of Urology, The First Affiliated Hospital of Anhui Medical University, Hefei, Anhui, China; 2 Department of Urology, The People’s Hospital of Chizhou, Chizhou, Anhui, China

**Keywords:** ApoB/Alb ratio, apolipoprotein B (ApoB), cross-sectional study, kidney stones, nephrolithiasis, NHANES, serum albumin

## Abstract

**Background:**

Kidney stones are widely considered a disease closely related to systemic metabolic disorders and systemic inflammation. The apolipoprotein B to albumin ratio (ApoB/Alb ratio) is a novel comprehensive biomarker that combines atherogenic lipid levels with systemic nutritional and inflammatory status. This study integrated data from two independent cohorts to explore the association between the serum ApoB/Alb ratio and the risk of kidney stones in adults.

**Methods:**

The Chinese single-center retrospective cohort included 741 subjects (223 kidney stone patients and 518 healthy controls) evaluated from March 2022 to September 2025. For external validation, 12,108 adult participants from the US National Health and Nutrition Examination Survey (NHANES) database (2007–2016) were included. Multivariate logistic regression analysis evaluated the independent association, while restricted cubic spline (RCS) smoothing curve fitting explored the dose-response relationship.

**Results:**

In the Chinese cohort, the serum ApoB/Alb ratio in the kidney stone group was significantly higher than in the control group (1.92 ± 0.52 vs. 1.84 ± 0.47, P = 0.048). After adjusting for comprehensive confounding factors, an elevated ApoB/Alb ratio was identified as a significant risk factor for kidney stones (OR = 3.26, 95% CI: 1.98–5.38). This significant positive association was highly consistent in the US NHANES validation cohort (OR = 1.30, 95% CI: 1.06–1.58). RCS analysis revealed a continuous positive linear dose-response relationship between the ApoB/Alb ratio and the prevalence of kidney stones in both cohorts. Furthermore, sensitivity and subgroup analyses demonstrated that this predictive value remains robust and is largely independent of traditional metabolic comorbidities such as hypertension and diabetes.

**Conclusion:**

As a comprehensive indicator of lipid metabolic disorders and inflammatory/nutritional imbalance, the ApoB/Alb ratio serves as a novel, highly sensitive, and reliable biomarker for predicting kidney stone risk.

## 1 Introduction

Kidney stones are one of the most common diseases of the urinary system. Their pathological feature is the imbalance of crystals and colloids in the urine, leading to the abnormal deposition of mineral salts in the kidneys (Weipu et al., 2022). The global incidence of kidney stones is increasing year by year. Epidemiological surveys in developed countries show that its prevalence has reached 5% to 15% (Finger et al., 2023). As a populous country, the incidence of kidney stones in China has also experienced significant growth. A recent national cross-sectional study reported that the prevalence of kidney stones in Chinese adults is approximately 6.4%, and the age of onset continues to show a younger trend (Guangyuan et al., 2022). A key clinical feature of this disease is the extremely high recurrence rate. About one-third of patients will experience a recurrence after their first episode (Rule et al., 2014), and the lifetime recurrence rate is even close to 50% (Ferraro et al., 2017; Siener, 2021). Recurrent stones not only bring severe pain, urinary tract obstruction, and renal function damage to patients (Ma et al., 2014) but also cause a huge medical economic burden (Hyams and Matlaga, 2014; Akram and Idrees, 2019). Therefore, identifying effective metabolic predictors and developing targeted prevention strategies are current priorities in urological research.

In recent years, with deepened research, kidney stones have been redefined as a systemic metabolic disease rather than a purely local urinary tract lesion (Ma et al., 2014). Abundant evidence shows that kidney stones are closely related to various chronic diseases, especially cardiovascular disease, metabolic syndrome, and chronic kidney disease (Reiner et al., 2011; Hsi et al., 2016). Although the specific mechanisms connecting kidney stones with cardiovascular comorbidities have not been fully elucidated, dyslipidemia is considered a key bridge between the two (Torricelli et al., 2014). Multiple studies have pointed out that dyslipidemia plays an important role in the formation of kidney stones, and this effect is partly related to other components of the metabolic syndrome (Torricelli et al., 2014; Hung et al., 2022; Gao et al., 2024).

Among many lipid and nutritional indicators, apolipoprotein B (ApoB) and serum albumin (Albumin) each play important roles in the pathophysiological process of kidney stones. On one hand, ApoB can more accurately reflect the total number of all atherogenic particles than traditional cholesterol indicators. Recent literature indicates that elevated ApoB levels or abnormal ApoB-related lipid ratios are significantly associated with the occurrence and high recurrence risk of kidney stones (Xia et al., 2023; Binbin et al., 2025). The underlying mechanism may be that high ApoB exacerbates oxidative stress and systemic inflammation in the body. This further promotes renal tubular epithelial damage and the retention of calcium oxalate crystals. On the other hand, serum albumin is not only a core indicator of the body’s nutritional reserves but also an important negative acute-phase reactant. It has significant antioxidant and anti-inflammatory effects. Recent epidemiological studies have confirmed that relatively low serum albumin levels (e.g., manifesting as a low albumin/globulin ratio) are closely related to an increased probability of developing kidney stones. This reflects the role of chronic inflammation and malnutrition in the pathogenesis of stones (Chen et al., 2025).

Previous studies mostly focused on single parameters in isolation. However, considering the highly complex cascade of kidney stone formation—which critically involves oxidative stress, renal tubular injury, inflammation, and crystal nucleation—combining ApoB (representing atherogenic and pro-inflammatory lipid burden) with albumin (representing anti-inflammatory, antioxidant, and physical crystal-defense capacity) into the ApoB/Alb ratio provides a more holistic biological rationale. An elevated ApoB/Alb ratio signifies a specific pathogenic microenvironment where ApoB-driven lipotoxicity and oxidative stress overwhelm the albumin-mediated macromolecular steric hindrance against crystal nucleation and aggregation (Liu et al., 2006; Baumann and Affolter, 2014; Farmanesh et al., 2014; Liu et al., 2017; Pan, 2022; Wen et al., 2025). Nevertheless, direct clinical evidence regarding the relationship between the ApoB/Alb ratio and the risk of kidney stones remains insufficient.

This study aims to systematically evaluate the association between the serum ApoB/Alb ratio and the prevalence of kidney stones in adult populations. This will be done through two independent population cohorts: a Chinese single-center clinical cohort and an external validation cohort from the US National Health and Nutrition Examination Survey (NHANES) database. Through this cross-regional and cross-ethnic dual-cohort validation study, we expect to provide a more universal epidemiological perspective for the comprehensive metabolic risk assessment of kidney stones. We also hope to offer valuable reference indicators for developing clinical prevention and intervention strategies.

## 2 Study population and design

This study is a dual-center, cross-regional cross-sectional study. It aims to explore the association between the serum apolipoprotein B to albumin ratio (ApoB/Alb ratio) and the risk of developing kidney stones.

### 2.2 Chinese single-center cohort

**Figure 1:**
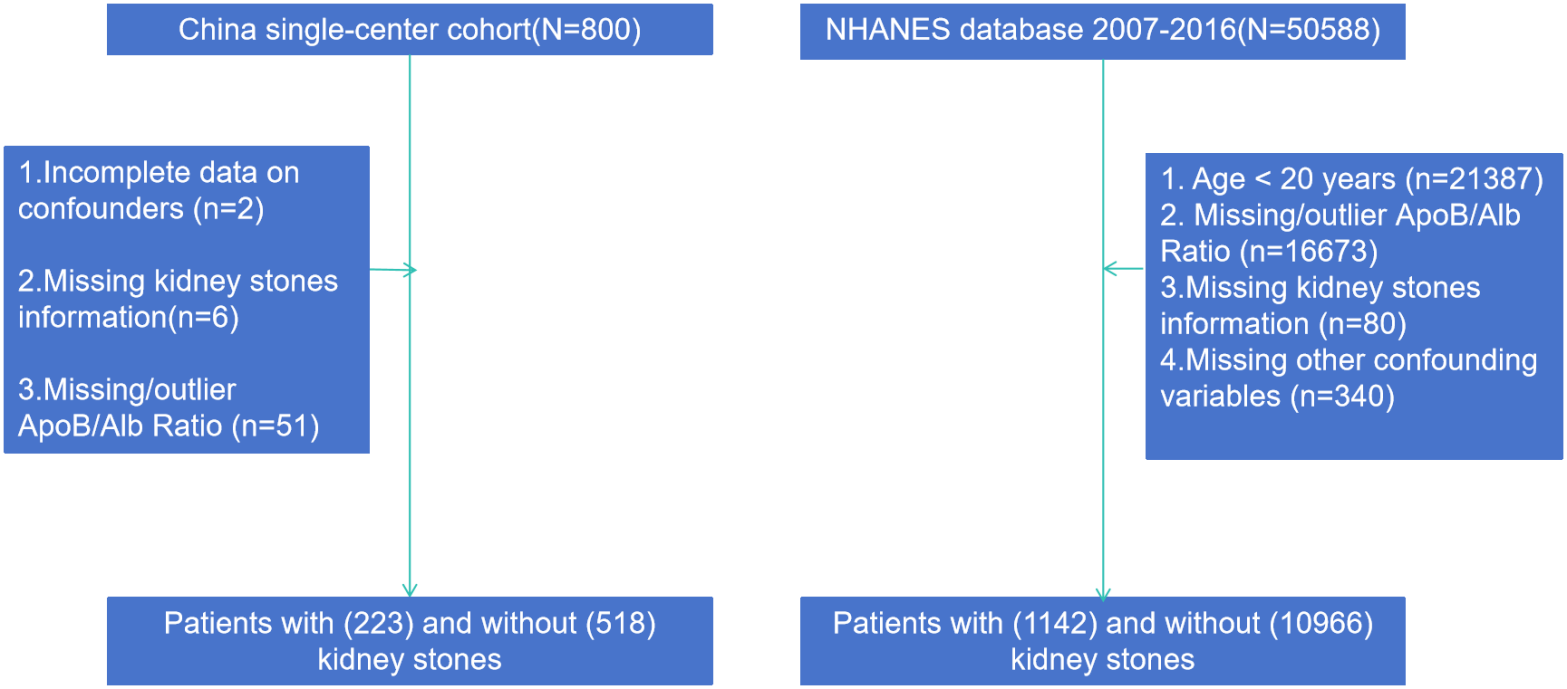
Flowchart of participant selection.

We retrospectively analyzed the data of subjects diagnosed at the Department of Urology, First Affiliated Hospital of Anhui Medical University between March 2022 and September 2025. The study was approved by the Ethics Committee of the First Affiliated Hospital of Anhui Medical University. All procedures followed the Declaration of Helsinki and its subsequent amendments. We initially screened 800 patients who underwent surgical intervention for kidney stones and healthy controls who were examined at the physical examination center during the same period. Exclusion criteria included (Weipu et al., 2022): missing data on key confounding factors (including age, sex, body mass index, hypertension, diabetes, blood urea nitrogen, serum uric acid, total cholesterol, triglycerides, serum calcium, fasting blood glucose, systemic immune-inflammation index, and serum creatinine) (Finger et al., 2023); presence of anatomical abnormalities such as a solitary kidney or horseshoe kidney (Guangyuan et al., 2022); missing apolipoprotein B or albumin measurements or the presence of extreme outliers. Finally, 741 subjects (223 stone patients and 518 controls) were included in the analysis (**Figure 1**).

### 2.3 US NHANES cohort

For external validation, this study integrated data from the 2007–2016 US National Health and Nutrition Examination Survey (NHANES). This project utilized a multi-stage, stratified probability sampling design. After excluding participants lacking kidney stone questionnaire records or key data on lipid metabolism, a total of 12,108 adult participants were finally included. This included 1,142 individuals who reported having kidney stones (**Figure 1**).

### 2.4 Data collection and laboratory measurements

#### 2.4.1 Data acquisition for the Chinese cohort

Demographic characteristics (age, sex, BMI) and clinical history (hypertension, diabetes) of the subjects were collected through the hospital’s electronic medical record system. Fasting venous blood samples were collected and processed using an automated assembly line. Serum ApoB and albumin levels were quantified using a Roche Cobas 6000 biochemical analyzer (Roche Diagnostics, Mannheim, Germany). Specifically, ApoB was measured via an immunoturbidimetric assay using proprietary Roche reagents calibrated against the WHO/IFCC reference standard. Serum albumin was measured using the bromocresol green (BCG) colorimetric method. Strict daily calibration and quality-control procedures were implemented, maintaining inter-assay and intra-assay coefficients of variation below 5%.

#### 2.4.2 Data acquisition for the NHANES cohort

Laboratory measurements adopted standardized protocols. They were completed through the Hitachi 704/717 or Roche Modular P automated biochemical analysis systems. Specifically, apolipoprotein B and related lipid profiles were quantified using immunonephelometric or immunoturbidimetric assays, as well as standard enzymatic methods. Serum albumin was measured primarily using the bromocresol purple (BCP) or bromocresol green (BCG) digital endpoint method on automated multiparameter chemistry analyzers. All laboratory platforms underwent rigorous standardization and calibration per the Centers for Disease Control and Prevention (CDC) guidelines. Detailed testing procedures, quality control plans, and analytical methodologies remain publicly available on the official NHANES website.

#### 2.4.3 Common biochemical indicators

These covered blood urea nitrogen (BUN), serum uric acid (UA), total cholesterol (TC), triglycerides (TG), serum calcium (Ca), fasting blood glucose (FPG), and serum creatinine (Scr). In addition, the systemic immune-inflammation index (SII = neutrophils × platelets/lymphocytes) was calculated to control for potential inflammatory effects (Di et al., 2023).

### 2.5 Definitions of variables

#### 2.5.1 Primary exposure variable

The apolipoprotein B to albumin ratio (ApoB/Alb ratio) is defined as the ratio of serum apolipoprotein B (mg/dl) to serum albumin (g/L). This indicator combines dual information reflecting pro-lithogenic lipid levels and anti-inflammatory nutritional status.

#### 2.5.2 Outcome variable

The Chinese cohort used computed tomography (CT) or B-mode ultrasound as the gold standard for diagnosing kidney stones. The NHANES cohort relied on a standardized self-report questionnaire (“Have you ever been told by a doctor that you have kidney stones?”).

### 2.6 Statistical analysis

All statistical analyses were performed using R language (version 4.2.2) and Empower software.

To address the issue of missing values in the datasets, we adopted a complete case analysis method (listwise deletion). For the Chinese single-center cohort study, the proportion of missing data was very low. Among the initially screened 800 subjects, 59 (approximately 7.4%) were excluded due to incomplete clinical confounding factors or unmeasurable apolipoprotein B (ApoB) and albumin levels. Given the relatively low missing rate, the data was considered missing completely at random (MCAR). Therefore, performing a complete case analysis is statistically adequate without introducing significant selection bias. Similarly, for the NHANES dataset, the large-scale exclusion of participants was mainly due to the complex survey design (e.g., specific laboratory test items were only conducted on predetermined subsamples), rather than due to patient non-compliance. Thus, the final regression models only included participants with complete and valid data for all necessary variables (including self-reported kidney stone history, target ApoB and albumin parameters, and basic demographic characteristics). To account for the complex, multistage, stratified probability sampling design of the NHANES database, all statistical analyses for the US cohort (including descriptive statistics and multivariable logistic regression models) strictly incorporated the appropriate survey weights, primary sampling units (PSUs), and stratification variables. This approach aligns with the Centers for Disease Control and Prevention (CDC) analytical guidelines, ensuring that standard errors are accurately calculated and the generated estimates are nationally representative. Furthermore, to ensure optimal analytical robustness, outliers in the ApoB/Alb ratio were defined as values below Q1 – 1.5 × IQR or above Q3 + 1.5 × IQR (where Q1 and Q3 are the first and third quartiles, respectively, and IQR is the interquartile range), and were subsequently excluded from the final analysis.

#### 2.6.1 Descriptive statistics

Normally distributed continuous variables were expressed as mean ± standard deviation (SD). T-tests were used for comparisons between groups. Non-normally distributed variables used the median (interquartile range) and non-parametric tests. Categorical variables were expressed as frequencies and percentages (%), and chi-square tests were used.

To rule out multicollinearity, collinearity diagnostics were performed for all covariates in the fully adjusted model. Variance inflation factors (VIFs) less than 5 were considered indicative of no significant multicollinearity. Regression model construction: Multivariate logistic regression analysis was used to calculate the odds ratios (OR) and 95% confidence intervals (CI) for the risk of kidney stones associated with the ApoB/Alb ratio. The models were constructed as follows:

Model 1: No covariates were adjusted.
Model 2: Adjusted for sex and age.
Model 3: Adjusted for all relevant confounding factors, including BMI, hypertension, diabetes, BUN, UA, TC, TG, Ca, FPG, SII, and creatinine.
Model 4 (Sensitivity analysis): Based on the fully adjusted model, populations with hypertension or diabetes were excluded to evaluate the independent robustness of the results.

#### 2.6.2 Dose-response relationship

Restricted Cubic Splines (RCS) and smoothing curve fitting were used to explore the continuous association between the ApoB/Alb ratio and the probability of having stones. Specifically, RCS is a flexible statistical framework used to model potential nonlinear dose-response relationships by fitting piecewise cubic polynomials across specified intervals (knots) of the continuous predictor. We utilized RCS to visually and statistically determine whether the risk of kidney stones increases linearly or non-linearly with an elevated ApoB/Alb ratio. Non-linear testing was conducted through a log-likelihood ratio test.

#### 2.6.3 Subgroup analysis

Stratified analyses were performed based on age (20–54 years vs. 55–85 years), sex, and the presence of hypertension and diabetes. This was to verify the consistency of the association across populations with different characteristics. To evaluate potential effect modification, subgroup analyses were conducted, and formal interaction testing was performed by including cross-product terms in the regression models to calculate the P for interaction.

## 3 Results

### 3.1 Comparison of baseline characteristics of the study population

This study included two independent cohorts: the Chinese single-center clinical cohort (n=741) and the US NHANES validation cohort (n=12,108).

Chinese cohort (**Table 1**): Among the 741 subjects, there were 223 kidney stone patients. The ApoB/Alb ratio in the stone group was significantly higher than that in the non-stone group (1.92 ± 0.52 vs. 1.84 ± 0.47, P = 0.048). In addition, the stone group had higher serum uric acid (UA) levels (P = 0.023) and lower fasting blood glucose (FPG) levels (P = 0.03). There were no significant differences between the two groups in age, BMI, blood urea nitrogen (BUN), total cholesterol (TC), triglycerides (TG), serum calcium, creatinine, SII index, sex, and the prevalence of hypertension and diabetes (all P > 0.05).

**Table 1:** Baseline data for populations in local regions of China.

Characteristic	Non-stone formers	Stone formers	P-value
N	518	223	
Age (years)	50.09 ± 12.31	51.22 ± 11.77	0.248
BMI (kg/m2)	24.42 ± 3.95	24.37 ± 3.30	0.855
BUN (mmol/L)	5.80 ± 3.19	6.07 ± 2.67	0.266
UA (umol/L)	327.59 ± 90.61	344.61 ± 99.13	0.023
Cholesterol (mmol/L)	4.58 ± 0.91	4.47 ± 0.92	0.134
TG (mmol/L)	1.78 ± 1.62	1.66 ± 0.85	0.278
Ca (mmol/L)	2.48 ± 0.21	2.35 ± 0.16	0.325
FPG (mmol/L)	5.48 ± 1.59	5.23 ± 0.95	0.03
SII	525.72 ± 463.51	490.86 ± 374.59	0.321
Cre (μmol/L)	86.31 ± 97.62	95.08 ± 56.04	0.21
ApoB/Albumin	1.84 ± 0.47	1.92 ± 0.52	0.048
Gender (%)			0.372
Male	300 (57.92%)	137 (61.43%)	
Female	218 (42.08%)	86 (38.57%)	
High Blood Pressure (%)			0.543
No	353 (68.15%)	157 (70.40%)	
Yes	165 (31.85%)	66 (29.60%)	
Diabetes (%)			0.207
No	424 (81.85%)	191 (85.65%)	
Yes	94 (18.15%)	32 (14.35%)	

BMI, body mass index; BUN, blood urea nitrogen; UA, uric acid; TC, total cholesterol; TG, triglycerides; Ca, serum calcium; FPG, fasting plasma glucose; SII, systemic immune-inflammation index; Scr, serum creatinine; ApoB/Albumin, apolipoprotein B to albumin ratio.

US NHANES cohort (**Table 2**): This cohort included a total of 12,108 subjects, of which 1,142 were in the stone group. The ApoB/Alb ratio in the stone group was also significantly higher than in the non-stone group (2.20 ± 0.56 vs. 2.14 ± 0.57, P < 0.001). Furthermore, the stone group had significantly higher levels of age, BMI, BUN, serum uric acid, blood glucose, triglycerides, and creatinine than the non-stone group (all P < 0.001). The prevalence of hypertension and diabetes was also significantly higher in the stone group (P < 0.001).

**Table 2:** Baseline population data from the US NHANES database.

Characteristic	Non-stone formers	Stone formers	P-value
N	10966	1142	
Age (years)	49.06 ± 17.64	56.03 ± 16.12	<0.001
BMI (kg/m2)	28.81 ± 6.78	30.39 ± 6.90	<0.001
BUN (mmol/L)	4.76 ± 2.08	5.23 ± 2.31	<0.001
Ca (mmol/L)	2.34 ± 0.09	2.34 ± 0.10	0.207
Cholesterol (mmol/L)	4.96 ± 1.02	4.90 ± 1.03	0.082
Cre (μmol/L)	77.94 ± 37.91	82.50 ± 56.59	<0.001
GLU (mmol/L)	5.67 ± 1.88	6.09 ± 2.20	<0.001
TG (mmol/L)	1.42 ± 1.10	1.57 ± 1.01	<0.001
UA (umol/L)	325.27 ± 84.89	334.39 ± 86.83	<0.001
SII	511.55 ± 321.23	563.62 ± 879.78	<0.001
ApoB/Albumin	2.14 ± 0.57	2.20 ± 0.56	<0.001
Gender (%)			<0.001
Male	5240 (47.78%)	632 (55.34%)	
Female	5726 (52.22%)	510 (44.66%)	
High Blood Pressure (%)			<0.001
Yes	3854 (35.14%)	570 (49.91%)	
No	7112 (64.86%)	572 (50.09%)	
Diabetes (%)			<0.001
Yes	1274 (11.62%)	244 (21.37%)	
No	9692 (88.38%)	898 (78.63%)	

BMI, body mass index; BUN, blood urea nitrogen; UA, uric acid; TC, total cholesterol; TG, triglycerides; Ca, serum calcium; FPG, fasting plasma glucose; SII, systemic immune-inflammation index; Scr, serum creatinine; ApoB/Albumin, apolipoprotein B to albumin ratio.

### 3.2 Independent association between the apoB/alb ratio and kidney stone risk (regression analysis)

By constructing multivariate logistic regression models, we evaluated the impact of the ApoB/Alb ratio on stone risk in the two populations (see **Table 3**):

**Table 3:** Association between ApoB/albumin and kidney stone risk: multivariable logistic regression analysis in Chinese and NHANES populations.

Characteristic	Model 1 OR (95% CI)	Model 2 OR (95% CI)	Model 3 OR (95% CI)	Model 4 OR (95% CI)
China data				
ApoB/Albumin	1.37 (0.99, 1.89)	1.36 (0.98, 1.89)	3.26 (1.98, 5.38)	3.28 (1.62, 6.62)
Categories				
Low (0.70-1.61)	1	1	1	1
Middle (1.61-2.02)	0.98 (0.66, 1.45)	0.97 (0.65, 1.44)	1.40 (0.90, 2.20)	1.71 (0.97, 3.02)
High (2.02-3.21)	1.38 (0.94, 2.02)	1.37 (0.93, 2.01)	3.02 (1.73, 5.26)	2.14 (1.03, 4.45)
NHANES data				
ApoB/Albumin	1.20 (1.08, 1.34)	1.14 (1.02, 1.27)	1.30 (1.06, 1.58)	1.03 (1.01, 1.04)
Categories				
Low (0.60-1.86)	1	1	1	1
Middle (1.86-2.37)	1.27 (1.09, 1.49)	1.19 (1.02, 1.39)	1.27 (1.07, 1.52)	1.20 (0.92, 1.57)
High (2.37-3.73)	1.32 (1.13, 1.54)	1.22 (1.04, 1.42)	1.38 (1.10, 1.74)	1.21 (0.85, 1.73)

Model 1= No covariates were adjusted.

Model 2= Model 1+ age, gender

Model 3 was adjusted for all covariates in **Table 1** or **Table 2**.

Model 4 was adjusted for the same variables as Model 3 and excluded individuals with hypertension and diabetes.

#### 3.2.1 Chinese data

In the fully adjusted model (Model 3, adjusting for age, sex, BMI, hypertension, diabetes, blood uric acid, blood glucose, creatinine, etc.), the ApoB/Alb ratio showed a significant positive association with the risk of kidney stones (OR = 3.26, 95% CI: 1.98–5.38). Tertile analysis showed that the stone risk in the high-level group (High) was 3.02 times that of the low-level group (Low) (95% CI: 1.73–5.26).

#### 3.2.2 NHANES data

The validation results showed a high degree of consistency. In the fully adjusted model (Model 3), for each unit increase in the ApoB/Alb ratio, the risk of kidney stones increased by 30% (OR = 1.30, 95% CI: 1.06–1.58). The risk in its highest tertile group was also significantly elevated (OR = 1.38, 95% CI: 1.10–1.74).

### 3.3 Smoothing curve fitting and dose-response relationship

**Figure 2:**
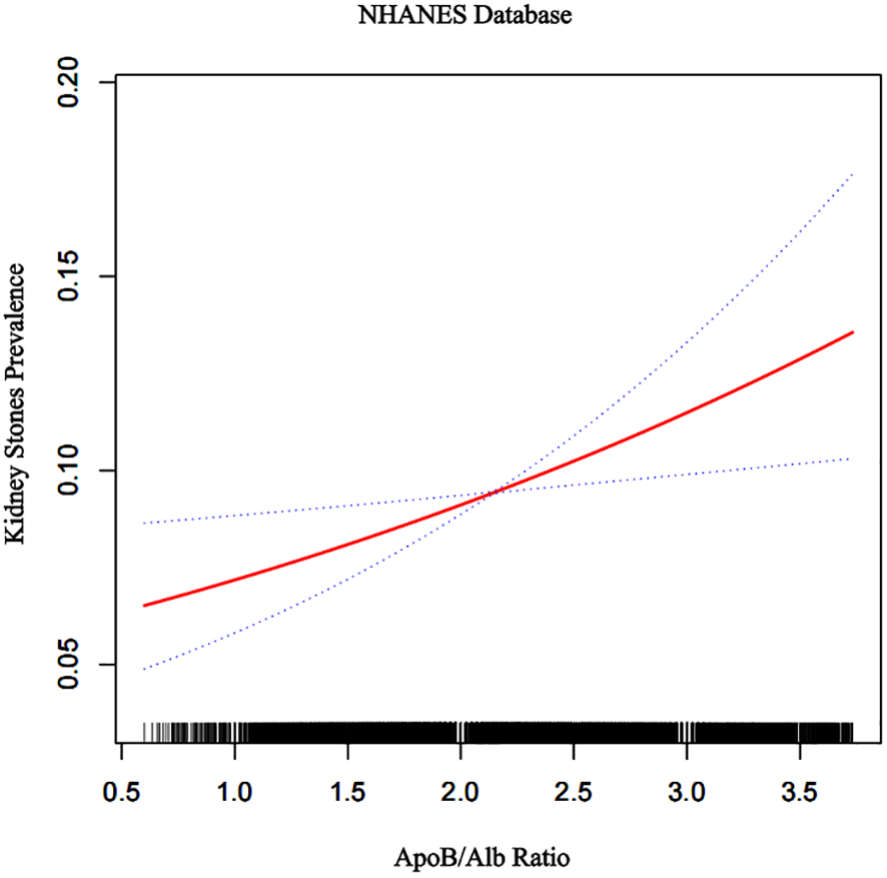
Dose–response relationship between ApoB/Alb ratio and kidney stone prevalence in the NHANES cohort.

**Figure 3:**
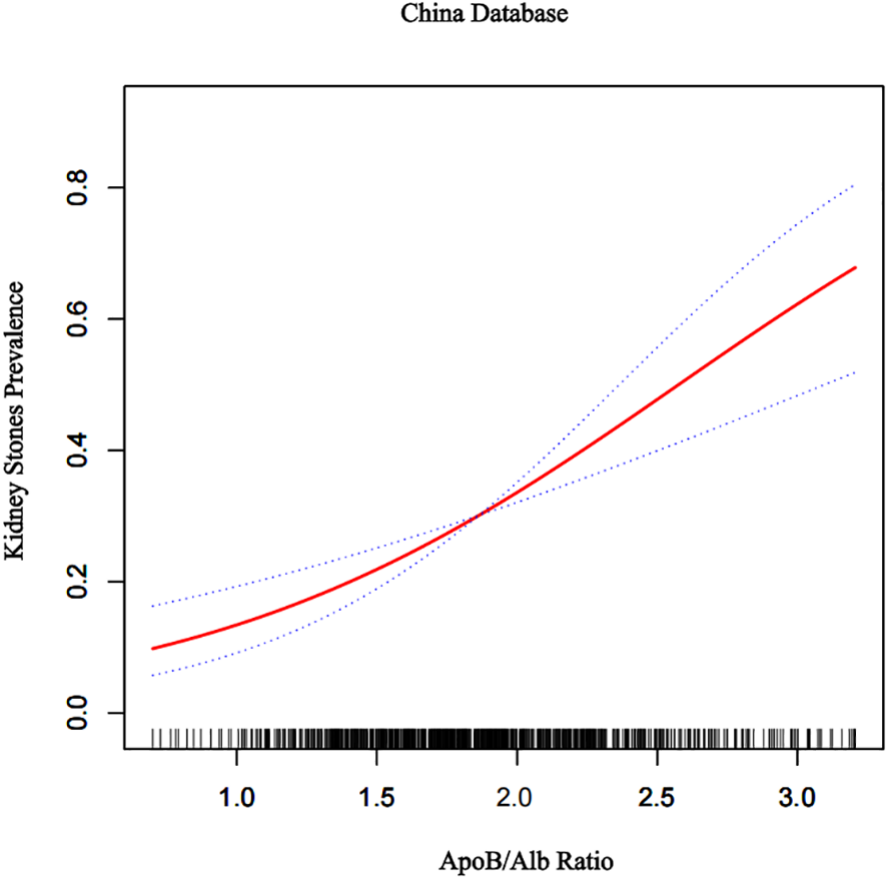
Dose–response relationship between ApoB/Alb ratio and kidney stone prevalence in the Chinese single-center cohort.

Through restricted cubic spline (RCS) analysis, the non-linear relationship between the ApoB/Alb ratio and kidney stone risk was further explored. The results showed that in both the Chinese cohort and the US NHANES cohort, the ApoB/Alb ratio and the prevalence of kidney stones exhibited a clear positive dose-response relationship (**Figures 2**, **3**). The fitted curves indicated that as the ApoB/Alb ratio continued to increase, the risk of kidney stones steadily rose, and no significant turning point was observed (non-linear test P > 0.05).

### 3.4 Subgroup analysis

Stratified analysis further verified the robustness of this association across populations with different clinical characteristics (see **Table 4**):

**Table 4:** Stratified analysis of the association between ApoB/albumin and kidney stone risk.

Characteristic	Model 1 OR (95% CI)	Model 2 OR (95% CI)	Model 3 OR (95% CI)	P for interaction
China data				
Stratified by age (years)				0.81
20-54	1.40 (0.94, 2.10)	1.42 (0.95, 2.13)	3.44 (1.86, 6.35)	
55-85	1.29 (0.75, 2.22)	1.29 (0.75, 2.23)	3.58 (1.39, 9.17)	
Stratified by gender				0.77
Male	1.33 (0.87, 2.05)	1.33 (0.86, 2.04)	3.87 (1.98, 7.55)	
Female	1.47 (0.90, 2.41)	1.39 (0.84, 2.31)	3.40 (1.44, 8.01)	
Stratified by hypertension				0.25
No	1.22 (0.82, 1.80)	1.18 (0.79, 1.76)	4.25 (2.25, 8.02)	
Yes	1.83 (1.02, 3.30)	1.84 (1.02, 3.33)	2.63 (1.04, 6.64)	
Stratified by diabetes				0.12
No	1.27 (0.88, 1.83)	1.24 (0.86, 1.79)	2.89 (1.63, 5.11)	
Yes	2.50 (1.14, 5.48)	2.59 (1.17, 5.75)	6.90 (2.01, 23.63)	
NHANES data				
Stratified by age (years)				0.001
20-54	1.41 (1.21, 1.64)	1.41 (1.21, 1.64)	1.18 (0.87, 1.58)	
55-85	0.96 (0.83, 1.12)	1.02 (0.87, 1.19)	1.39 (1.06, 1.81)	
Stratified by gender				0.29
Male	1.14 (0.99, 1.32)	1.13 (0.97, 1.31)	1.37 (1.04, 1.80)	
Female	1.29 (1.10, 1.50)	1.20 (1.02, 1.40)	1.16 (0.87, 1.55)	
Stratified by hypertension				0.89
No	1.02 (0.87, 1.19)	1.07 (0.91, 1.26)	1.30 (0.99, 1.70)	
Yes	1.31 (1.13, 1.51)	1.15 (0.99, 1.34)	1.24 (0.93, 1.65)	
Stratified by diabetes				0.78
No	1.17 (0.93, 1.47)	1.31 (1.03, 1.66)	1.49 (0.96, 2.33)	
Yes	1.21 (1.07, 1.37)	1.10 (0.97, 1.24)	1.26 (1.01, 1.57)	

#### 3.4.1 Chinese cohort

The positive association between ApoB/Alb and stone risk was established in both the 20–54 years (OR = 3.44) and 55–85 years (OR = 3.58) age groups. Although the odds ratios were numerically higher in male subjects (OR = 3.87, 95% CI: 1.98–7.55), non-hypertensive patients (OR = 4.25), and diabetic patients (OR = 6.90), formal interaction tests did not establish significant effect modification across age, sex, hypertension, or diabetes strata in this cohort (all P for interaction > 0.05).

#### 3.4.2 NHANES cohort

The association was statistically significant in the subgroups of 55–85 years (OR = 1.39), males (OR = 1.37), and diabetic patients (OR = 1.26). Notably, a significant age interaction was observed in this cohort (P for interaction = 0.001), suggesting that the risk association may be modified by age.

### 3.5 Sensitivity analysis

To verify whether this independent association is interfered with by common metabolic comorbidities, we excluded subjects with hypertension and/or diabetes in the Chinese cohort (see **Table 3**, Model 4). In this relatively healthy subject subpopulation, the positive association between the ApoB/Alb ratio and the risk of kidney stones remained highly significant (OR = 3.28, 95% CI: 1.62–6.62). This indicates that the predictive role of the ApoB/Alb ratio for stone risk is largely independent of the effects of hypertension and diabetes.

## 4 Discussion

By integrating large-sample data from the Chinese single-center clinical cohort and the US NHANES database, this study systematically explored and validated the association between the serum ApoB/Alb ratio and the risk of developing kidney stones. The study results showed that after adjusting for age, sex, body mass index (BMI), and specific confounding factors (including hypertension, diabetes, blood urea nitrogen, serum uric acid, total cholesterol, triglycerides, serum calcium, fasting blood glucose, systemic immune-inflammation index, and serum creatinine), a higher ApoB/Alb ratio showed a strong positive correlation with an increased risk of kidney stones. Notably, the effect size (odds ratio) of the ApoB/Alb ratio increased substantially from the unadjusted model to the fully adjusted model. This phenomenon suggests the presence of negative confounding (suppressor variables) among the clinical covariates—such as age, BMI, or specific metabolic indices. When these covariates are omitted, the true independent association is masked. Furthermore, collinearity diagnostics confirmed no multicollinearity among the adjusted covariates (all VIFs < 5). Given that our sample size (223 positive events) comfortably exceeded the conventional rule of thumb of 10 events per variable for the 11 covariates, adjusting for this comprehensive set of confounders minimizes omitted variable bias and more accurately reflects the true independent association between the ApoB/Alb ratio and kidney stones. Additionally, restricted cubic spline (RCS) analysis further revealed that a significant and continuous positive dose-response relationship existed between the ApoB/Alb ratio and stone risk in two distinctly different ethnic and geographical populations. Our findings in the US population align with and further extend the recent surge of epidemiological evidence from NHANES, which underscores the critical involvement of lipid metabolic disorders in kidney stone development (He et al., 2024; Xinyi et al., 2025).This series of epidemiological evidence collectively indicates that the ApoB/Alb ratio not only reflects the body’s lipid metabolic disorder. It is also an extremely sensitive comprehensive biomarker for evaluating the risk of kidney stone incidence.

This study selected the ApoB/Alb ratio as the core research object. Its scientific basis lies in the fact that this indicator can accurately capture the imbalance between the two key physiological microenvironments within the body that promote and inhibit stone formation (Wen et al., 2025). Apolipoprotein B (ApoB) is the core structural protein of all atherogenic lipid particles. Its abnormal accumulation in the renal microvascular system is considered a key initiating factor causing local “lipotoxicity” in the kidneys (Pan, 2022). Pathophysiologically, high levels of pathogenic lipids are highly prone to being converted into oxidized lipoproteins through oxidative modification. These oxidative products have been confirmed to induce strong oxidative stress in renal tubular epithelial cells by activating inflammatory pathways. This leads to cell membrane lipid peroxidation and a massive accumulation of reactive oxygen species (ROS) (Pan, 2022; Wen et al., 2025). Conversely, serum albumin is not only a core indicator measuring the body’s overall nutritional reserves. It is also a crucial physical defense barrier against crystallization in the urine microenvironment. Recent physical chemistry and crystallization kinetics studies have confirmed that macromolecules like serum albumin can specifically adsorb and “pin” onto the spiral dislocation steps on the surface of calcium oxalate crystals through strong protein-crystal interface interactions (Farmanesh et al., 2014). This molecular-level spatial steric hindrance effect can directly coat tiny crystals. It not only significantly inhibits the initial kinetics of crystal nucleation but also strongly blocks the layer-by-layer growth and large-scale aggregation of crystals from a physical space perspective (Liu et al., 2006; Farmanesh et al., 2014). Therefore, when the body’s albumin levels relatively decrease (i.e., the ApoB/Alb ratio increases), this molecular-level crystal defense mechanism in the renal microenvironment is significantly weakened. This provides favorable conditions for the uncontrolled growth of supersaturated crystals. Consequently, an elevated ApoB/Alb ratio reflects a microenvironment where pro-inflammatory and pro-oxidative factors overwhelm natural crystal defenses. This lipid-protein metabolic imbalance directly accelerates early microscopic stone-forming cascades (Liu et al., 2017). Frontier pathological research shows that lipotoxicity triggered by abnormal lipid deposition will trigger macrophage infiltration and chronic inflammatory responses in the renal interstitium (Liu et al., 2017; Wen et al., 2025). Persistent inflammation and a highly oxidative microenvironment will irreversibly destroy the apical membrane integrity of renal tubular epithelial cells. This prompts the exposure of a large number of crystal adhesion molecules, such as osteopontin and hyaluronic acid, on the surface of damaged cells (Baumann and Affolter, 2014). These exposed matrix macromolecules act as “anchor points” for crystallization. They greatly increase the retention time of supersaturated calcium oxalate microcrystals in the renal tubular lumen, thereby promoting the formation of Randall’s plaques and the final growth of solid stones (Baumann and Affolter, 2014; Liu et al., 2017).

In the comparative analysis of the results from the two independent cohorts, we observed that the strength of the risk association in the Chinese population cohort was numerically higher than in the NHANES cohort (Chen et al., 2023). Notably, the baseline ApoB/Alb ratio was substantially higher in the NHANES cohort than in the Chinese cohort. This cross-population discrepancy is likely driven by multiple factors, including differences in demographic characteristics, ethnicity, dietary patterns (such as a higher intake of ultra-processed foods), and a higher prevalence of baseline obesity (Juul et al., 2018; Wang et al., 2021). Furthermore, it is critical to acknowledge methodological differences: the NHANES dataset frequently utilizes measurements related to small dense LDL (sdLDL) to assess atherogenic burden, whereas our Chinese cohort measured ApoB directly. This methodological variance between sdLDL and direct ApoB assays likely contributes significantly to the baseline differences in the calculated ratios between the two populations (Ivanova et al., 2017). This cross-population heterogeneity in effect size may reflect differences in sensitivity to lipid metabolic disorders caused by different environments, dietary structures, and genetic backgrounds (Wong et al., 2016; Chen et al., 2023). Chinese kidney stone patients are often accompanied by specific dietary changes towards high sodium and high animal protein (Guohua et al., 2017). Such environmental stress brought about by dietary changes can significantly alter the local physicochemical microenvironment of the kidneys (Ferraro et al., 2020). This greatly amplifies the ‘lipotoxic’ damage to the renal tubular epithelium caused by lipid metabolic imbalance (dyslipidemia) locally and its related pathogenic effects (Torricelli et al., 2014). Beyond these environmental and dietary factors, this cross-cohort heterogeneity in effect size is also fundamentally driven by the differences in study design and the severity of the disease captured. The Chinese single-center cohort is a hospital-based surgical cohort, enrolling patients who required surgical intervention, which inherently represents a population with severe, symptomatic, or recurrent kidney stones. In contrast, the NHANES database is a community-based cross-sectional survey where stone history relies on self-reporting, inevitably encompassing a large proportion of asymptomatic, mild, or historical stone formers. Crucially, this fundamental difference in outcome ascertainment—CT/ultrasound-confirmed diagnosis in the Chinese cohort versus self-report questionnaires in NHANES—introduces substantial differential misclassification. Self-reported stone history is highly susceptible to recall bias. Furthermore, a significant number of asymptomatic stone formers in the NHANES population may be incorrectly categorized into the non-stone control group. Epidemiologically, such non-differential or differential misclassification tends to dilute the true association and attenuate the observed effect size (bias toward the null). Therefore, this misclassification bias likely serves as a primary driver for the attenuated odds ratio observed in the NHANES cohort (OR 1.30) compared to the rigorously imaging-confirmed Chinese cohort (OR 3.26). Therefore, the pronounced association observed in the Chinese cohort indicates that an elevated ApoB/Alb ratio is particularly sensitive and highly predictive for the development of severe, clinically significant stones requiring surgical treatment.

More importantly, in the sensitivity analysis excluding subjects with hypertension and diabetes, the positive correlation between the ApoB/Alb ratio and kidney stones not only persisted but demonstrated a remarkably strong association (OR = 3.28). This profound epidemiological finding shifts the paradigm of how we view this biomarker. It powerfully demonstrates that the ApoB/Alb ratio is not merely a byproduct or a late-stage manifestation of established systemic metabolic syndrome. Instead, it serves as an exceptionally sensitive early-warning biomarker. It can accurately identify individuals who appear clinically healthy (normotensive and normoglycemic) but are already in a ‘pre-metabolic disorder state’ with a highly lithogenic renal microenvironment.

The subgroup analysis data further revealed the epidemiological characteristics of this biomarker. Interestingly, the magnitude of the association (ORs) in specific subgroups (such as distinct age brackets and normotensive patients) appeared higher than the overall adjusted OR. Statistically, this phenomenon can be partially explained by the inherent non-collapsibility of odds ratios in multivariate logistic regression when strong covariates are adjusted. Clinically, this numerically suggests a potential effect modification: the lithogenic impact of an imbalanced ApoB/Alb ratio appears more prominent in patients without overlapping severe metabolic conditions (like hypertension). In populations where other major metabolic risk factors are absent, this specific lipid-protein imbalance becomes a critical risk factor for stone formation. Furthermore, the ApoB/Alb ratio showed a numerically higher association with stone risk in the male group than in females. However, it is important to note that formal interaction tests did not establish significant effect modification for sex or hypertension (all P_for interaction > 0.05), indicating that the fundamental risk association remains robust across these different populations.

This significant sexual dimorphism can be attributed to the dual antagonistic effects of sex hormones in regulating systemic lipid profiles and local renal physiology (Peerapen and Thongboonkerd, 2019; Gillams et al., 2021). Evidence at the molecular level shows that estrogen can not only upregulate hepatic clearance receptors to improve lipid metabolism. It can also directly reduce the deposition of urinary crystals by regulating related transporters locally in the kidneys (Peerapen and Thongboonkerd, 2019). Conversely, an androgenic environment may exacerbate local oxidative stress cascades. This makes men exhibit higher susceptibility to stones when facing the same degree of lipoprotein/albumin imbalance (Liang et al., 2014). From the perspective of clinical translation, this ratio relies on widely accessible and low-cost intravenous biochemical testing. Incorporating it into routine metabolic screening will greatly assist urologists in accurately identifying “pre-metabolic disorder” populations who have not yet suffered from typical cardiovascular diseases but are already at high risk of developing stones. This thereby provides a new time window for intervention.

Although this study enhanced the generalizability of its conclusions through a dual-cohort design, there are still several methodological limitations. First, the cross-sectional study design based on existing data cannot confirm a unidirectional causal relationship between an elevated ApoB/Alb ratio and kidney stone formation over a time span. Second, limited by the collection scope of the databases, this study lacks detailed infrared spectroscopic composition analysis data for the stones. This limits our in-depth exploration of the heterogeneous role of this lipid metabolism ratio in stones of different pathological subtypes (such as uric acid stones and calcium oxalate stones). Finally, the absence of 24-hour urine biochemical parameters (such as urinary calcium, urinary oxalate, and urinary citrate) means that we currently cannot precisely quantify how this systemic blood indicator affects the stone-forming microenvironment by altering the physicochemical properties of urine. Additionally, the marginally lower fasting blood glucose (FPG) levels observed in the Chinese stone group compared to healthy controls is likely an artifact of information bias introduced by clinical protocols: surgical stone patients undergo strict pre-operative fasting prior to blood collection. Future explorations urgently need to conduct prospective cohort studies with long-term follow-up. Combined with multi-omics and animal model technologies, we need to deeply decode the specific molecular pathways by which the ApoB/Alb ratio regulates the urinary tract stone-forming microenvironment.

## 5 Conclusion

In summary, this study, based on dual validation from a Chinese single-center clinical cohort and the US NHANES database, confirmed a significant and robust positive correlation between an elevated serum apolipoprotein B to albumin ratio (ApoB/Alb ratio) and an increased risk of kidney stones in adult populations. Moreover, the two exhibit a continuous positive dose-response trend. As a novel biomarker that comprehensively reflects the imbalance between the body’s atherogenic lipid burden and systemic nutritional defense reserves, an abnormally elevated ApoB/Alb ratio reveals the comprehensive suppression of physical crystal defense mechanisms by the pro-inflammatory and pro-oxidative microenvironment in the local kidneys.

In clinical practice, this ratio has the advantages of being convenient to detect and low in cost. Incorporating it into routine metabolic screening is expected to become an effective tool for evaluating the risk of kidney stone incidence. In particular, it can help clinicians accurately identify susceptible populations (especially the male group) who have not yet suffered from typical systemic metabolic diseases but are already in a “pre-metabolic disorder state” with a high risk of stones. In the future, it is urgent to conduct large-scale prospective follow-up cohort studies to confirm its causal association. Furthermore, combining multi-omics technologies is needed to deeply analyze the specific molecular pathways of this lipid-protein metabolic axis in regulating the urinary tract stone-forming microenvironment.

## Data Availability

The raw data supporting the conclusions of this article will be made available by the authors, without undue reservation.
